# Simulation-based economic evaluation of the Wolbachia method in Brazil: a cost-effective strategy for dengue control

**DOI:** 10.1016/j.lana.2024.100783

**Published:** 2024-06-03

**Authors:** Ivan Ricardo Zimmermann, Ricardo Ribeiro Alves Fernandes, Márcia Gisele Santos da Costa, Márcia Pinto, Henry Maia Peixoto

**Affiliations:** a Department of Public Health, University of Brasilia, Brasilia, Brazil; b Health Technology Assessment Unit, National Cancer Institute, Rio de Janeiro, Brazil; c Health Technology Assessment Unit, National Cardiology Institute, Rio de Janeiro, Brazil; d Fernandes Figueira Institute, Oswaldo Cruz Foundation, Rio de Janeiro, Brazil; e Center for Tropical Medicine, University of Brasilia, Brasilia, Brazil

**Keywords:** Wolbachia, Dengue, Cost-effectiveness, Brazil

## Abstract

**Background:**

Dengue virus (DENV) is an arbovirus transmitted by *Aedes aegypti* mosquitoes, which can cause severe conditions such as hemorrhagic fever and dengue shock syndrome. These conditions are associated with adverse social, clinical, and economic consequences in Brazil. Herein, the Wolbachia mosquito replacement method is a promising dengue control strategy.

**Methods:**

We estimated the economic impact of implementing the *Wolbachia* mosquito replacement method in seven Brazilian cities. A mathematical microsimulation model tracked nearly 23 million inhabitants over a 20-year period, considering the transitions between five different health states (susceptible, inapparent, outpatient, hospitalised and death). Direct costs included local dengue control programs, Wolbachia implementation and dengue care. Indirect costs related to death and productivity loss, as well as disability-adjusted life-years (DALY) averted were also considered.

**Findings:**

Without Wolbachia, the model projected 1,762,688 reported dengue cases over 20 years. Implementing the Wolbachia method would avert at least 1,295,566 dengue cases, resulting in lower costs and greater effectiveness in all simulated cities. On average, for every 1000 inhabitants followed for 20 years, the Wolbachia method yielded a cost difference of USD 538,233.68 (BRL 2,691,168.40) and averted 5.56 DALYs. Net monetary benefits (NMB) were positive in all seven cities, ranging from USD 110.72 (BRL 553.59) to USD 1399.19 (BRL 6995.95) per inhabitant. Alternative scenarios have also shown a favourable return on investment with a positive benefit-cost ratio (BCR).

**Interpretation:**

Wolbachia is likely a cost-effective strategy in the Brazilian context, consistent with international studies. Sensitivity analysis and alternative scenarios confirmed the robustness of the results.

**Funding:**

This study was funded by the Wellcome Trust under a grant (224459/Z/21/Z).

**Research in context**

**Evidence before this study**

Previous research has established the significant social, clinical, and economic consequences of dengue virus (DENV) transmission in Brazil. Severe conditions like dengue shock, severe bleeding, and severe organ impairment have been identified as significant causes of disability and mortality. In recent years, the implementation of the Wolbachia method has emerged as a promising and innovative dengue control strategy.

We searched PubMed (MEDLINE) and LILACS for studies reporting the cost-effectiveness of Wolbachia in dengue, in November 2023, considering the search terms “Wolbachia”, “dengue”, and “cost-effectiveness”. The search was supplemented with Google Scholar and references in identified papers. Based on these searches, 6 studies were identified, which assessed the economic impacts of the Wolbachia method in different settings. The studies have shown that short-term releases of *Aedes aegypti* mosquitoes infected with the wMel strain of Wolbachia can achieve stable introduction of the bacterium into local *Ae. aegypti* populations, resulting in reduced arboviral disease burden. Robust evidence has been published on the efficacy of this method, including a cluster-randomized trial in Indonesia, which demonstrated a protective efficacy of 77.1% and observational data in Colombia showing more than 95% reduction in dengue cases reported. Real-world data from Brazil also showed a 69% reduction in dengue incidence after introducing the Wolbachia strain into mosquito populations. Based on these estimates, previous economic evaluations have concluded the Wolbachia method is a cost-effective method for controlling dengue in the Brazilian state of Goias and other international contexts like Indonesia, Singapore, Thailand and Vietnam.

**Added value of this study**

This study aimed to estimate the cost-effectiveness of implementing the Wolbachia method in seven different cities in Brazil, filling a gap in knowledge regarding the economic impact of this strategy in the Americas. By using a mathematical microsimulation model, the study projected the trajectories of nearly 23 million inhabitants over 20 years, considering direct and indirect costs, as well as disability-adjusted life-years (DALY). The findings suggested that adding the implementation of the Wolbachia method to usual dengue control strategies would lead to a significant reduction in dengue cases, resulting in lower costs and greater effectiveness across all simulated cities. The study provided quantifiable economic indicators, such as net monetary benefits (NMB) and benefit-cost ratios (BCR), which are crucial for evaluating the feasibility and value of implementing this strategy.

**Implications of all the available evidence**

The results of this study contribute to the growing body of evidence supporting the cost-effectiveness of the Wolbachia method for dengue control, particularly in the Brazilian context. The findings align with international studies and confirm that the implementation of the Wolbachia method is a cost-effective strategy. The robustness of the results was further reinforced by sensitivity analysis and alternative scenarios. The study’s implications are relevant for policymakers and public health authorities, providing evidence-based support for the integration of the Wolbachia method into dengue control programs. By implementing this strategy, significant reductions in dengue cases and associated economic burdens can be achieved. These findings have the potential to inform future decisions regarding public policies on dengue control in Brazil and other dengue-affected regions.

## Introduction

Dengue virus (DENV) is one of the most important arboviruses transmitted by the *Aedes aegypti* mosquito, with substantial social, clinical and economic consequences in Brazil.^1^ While many DENV-infected individuals show mild or no symptoms, a significant number develop severe dengue, leading to hospitalization. The WHO defines severe dengue as plasma leakage, severe bleeding, or organ damage, all of which increase morbidity and mortality risk.^2^^,^^3^ In addition to clinical consequences, based on a Brazilian societal perspective, the estimated annual costs of dengue, including ambulatory, hospitalized, and fatal cases, have been estimated between USD 516.79 million (2009) and 1688.3 million (2013).^4^

In the context of dengue control strategies, the implementation of the Wolbachia method is a promising and innovative option. This strategy involves the infection of *Ae. aegypti* mosquitoes with Wolbachia (*w*Mel strain), a very common intracellular bacterium present in insects in nature. Once infected, female *Ae. aegypti* transmit the bacterium with high fidelity to their offspring via infected eggs and Wolbachia manipulates mosquito reproductive outcomes via a process called cytoplasmic incompatibility, which favours introgression of Wolbachia into the wild-type population. Furthermore, this particular Wolbachia strain has been shown to block virus transmission, therefore reducing arbovirus transmission.^5^ Robust evidence has been published about this method in recent years. For example, after enrolling 8144 participants, a cluster-randomized trial of Wolbachia deployment in Yogyakarta, Indonesia,^6^ demonstrated a protective efficacy of 77.1% (95% confidence interval, 65.3–84.9). In the context of Latin America, contiguous deployment of Wolbachia to date in 3 adjacent cities in the Aburra Valley, Colombia (3.3 million people, 135 km^2^) showed that dengue incidence was reduced by 95–97% during the 2–4 years following Wolbachia deployment, compared to the 10 years pre-intervention.^7^ These estimates were consistent with real-world Brazilian data showing that the introduction of the Wolbachia strain into *Ae. aegypti* mosquitoes in the city of Niteroi, Brazil, during 2017–2019, was associated with a 69% reduction in dengue incidence (95% CI, 54–79).^8^ Similarly, in areas of Rio de Janeiro where Wolbachia prevalence in *Ae. aegypti* exceeded 60% following releases in 2017–2019, the incidence of dengue was 71% lower (95% CI, 64–76) and the incidence of chikungunya was 23% lower (95% CI, 11–35%) when compared with areas of the city without Wolbachia.^9^

Brazil’s national dengue control program is mostly based on epidemiological and entomological surveillance and response activities, including conventional approaches to reducing mosquito populations through environmental and chemical control.^10^ However, the Wolbachia method is now being implemented in 5 Brazilian cities, and dengue vaccines are now available for specific groups in Brazil, with expected expansion in the coming years.^11^ Considering the importance of dengue burden and its consequences, we aimed to estimate the cost-effectiveness of the implementation of the Wolbachia method in seven different cities in Brazil to support future decisions on public policies on dengue control.

## Methods

Our study followed the current national^12^ and international methodological guidelines for health economic evaluation,^13^^,^^14^ as well as the latest version of the Consolidated Health Economic Evaluation Reporting Standards – CHEERS guidelines.^15^

### Study population, setting and location

**Fig. 1:**
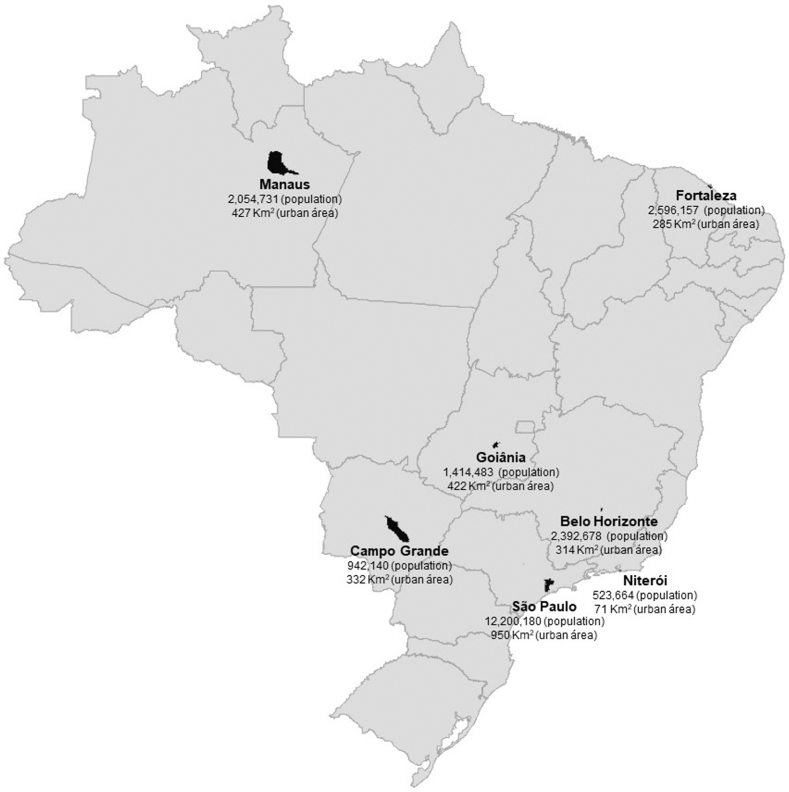
Geographic representation of the seven Brazilian cities included in the model.

The economic evaluation included estimates for the total population of seven municipal areas in Brazil identified as high priority to receive the Wolbachia method, based on population size and dengue disease burden: São Paulo, Fortaleza, Campo Grande, Goiania, Belo Horizonte, Manaus and Niteroi. These seven cities are located in different Brazilian geographic regions, with varying social and demographic characteristics, and with a combined population of almost 23 million inhabitants (Fig. 1).

### Comparators

The economic model considered the reference scenario of the routine activities carried out by public health authorities as part of the dengue control program, including human resources, surveillance infrastructure and educational campaigns. The alternative scenario considers the routine activities of the dengue control program and the addition of the Wolbachia method (short-term releases of Wolbachia-infected *Ae. aegypti*).^8^

### Perspective

The model adopted the Brazilian Society perspective, considering both direct and indirect costs.^12^

### Time horizon

Costs and consequences were estimated with a 20-year time horizon, starting from 2023 to 2042. The time horizon aimed to ensure complete coverage of at least one cycle of infection circulation for all dengue serotypes.^16^, ^17^, ^18^ Given the variability of these cycles, as well as their potential impact on outcomes, we considered alternative scenarios with shorter time horizons of 5 and 10 years.

### Discount rate

According to Brazilian guidelines,^12^ an annual discount rate of 5% was applied to costs. Regarding the fact that discounting substantially affects the cost-effectiveness of preventive interventions and programs with immediate cost, our model did not consider discounting health benefits. This decision is supported by recent publications from the World Health Organization (WHO),^19^ recommending the zero-discount rate for health benefits in these situations. Recognizing this as a point of methodological uncertainty, we ran our model in two alternative scenarios with discounts on costs and outcomes, as well as without any discount.

### Selection and measure of outcomes

We measured clinical benefits by DALYs averted, calculating the Incremental Cost-Effectiveness Ratio (ICER) for the Wolbachia method. Results were adjusted for population (per 1000 residents) to compare city-level outcomes. The official Brazilian cost-effectiveness threshold of BRL 120,000 (USD 24,000) per DALY for neglected disease technologies was adopted.^20^ We also computed Net Monetary Benefits (NMB) and Benefit-Cost Ratios (BCR) for Wolbachia, converting DALY benefits into monetary terms at this threshold.

### Valuation of outcomes

The DALY burden of dengue was calculated as the sum of the total years lived with disability (YLDs; morbidity component) and the years of life lost (YLLs) due to premature death (mortality component). The YLDs were estimated by the application of the disability weights (DWs), interpreted as the proportional reduction in good health due to an adverse health state, as estimated in the GBD study^21^(Table 1). The YLLs due to premature death were based on each age-specific expectancy of the standard life expectation adopted by the GBD against the age of death in the model.

**Table 1:** Summary of the model parameter values and sources for Brazilian cities.

Parameter	Description	Base case value (range)	Source
c_out_priv	Private outpatient treatment cost (BRL)	414.39 (200.00–900.00)	ANS; CMED
c_out_pub	Cost of public outpatient treatment (BRL)	367.01 (123.61–572.41)	BPS; SIGTAP
c_hosp_priv	Private Hospital Treatment Cost (BRL)	2344.32 (1875.46–2813.18)	Machado (2019)^22^
c_hosp_pub_nonsevere	Cost of public hospital treatment for non-severe dengue (BRL)	1327.88 (1062.30–1593.45)	SIH/DATASUS^a^
c_hosp_pub_severe	Cost of public hospital treatment for severe dengue (BRL)	3488.52 (2790.81–4186.22)	SIH/DATASUS^a^
c_program_dengue	Cost of Dengue Control Program (per inhabitant) (BRL)	34.37 (27.49–41.24)	Taliberti (2010)^23^; Santos (2015)^24^
c_wolbachia	Wolbachia method implementation incremental cost (per inhabitant) (BRL)	12.74 (12.74–35.75)	WMP (2023)
c_ind_death	Indirect cost of productivity loss due to premature death (BRL)	937,922.86 (750,338.29–1,125,507.43)	ISPER^25^^,^^a^
c_ind_out	Indirect cost of productivity loss due to outpatient treatment (BRL)	1039.91 (831.93–1247.90)	ISPER^25^^,^^a^
c_ind_hosp	Indirect cost of productivity loss due to inpatient treatment (BRL)	2244.02 (1795.21–2692.82)	ISPER^25^^,^^a^
multiplication_factor	Case expansion multiplier due to underreport in SINAN	12 (9.8–14.9)	Silva (2016)^26^
p_inapparent	Proportion of inapparent infections (asymptomatic or self-care)	0.6060 (0.4848–0.7272)	Azeredo (2017)^27^; Vazquez-Prokopec (2023)^28^
p_outpatient	Proportion of outpatient cases (symptomatic)	0.3536 (0.28288–0.42432)	SINAN/DATASUS^a^
p_hospital	Proportion of hospitalized cases	0.0404 (0.03232–0.04848)	SINAN/DATASUS^a^
p_self_care	Proportion of inapparent infections having symptoms (self-care)	0.125 (0.0–0.125)	Aguiar (2021)^29^
p_nonsevere	Proportion of severe dengue in hospitalized cases	0.1466 (0.11728–0.17592)	SIH/DATASUS^a^
p_death_overall	Probability of general death according to age	0.0023 (0.00184–0.00276)	IBGE, 2021^a^
p_death_hospital	Probability of death in hospitalized cases (case-fatality rate)	0.0165 (0.0132–0.0198)	SIH/DATASUS^a^
d_weight_dengue_severe	Disability weight in severe cases of dengue	0.133 (0.1064–0.1596)	GBD (2019)^30^
d_weight_dengue_moderate	Disability weight in moderate cases of dengue	0.051 (0.0408–0.0612)	GBD (2019)^30^
d_weight_fatigue	Disability weight in cases of post-dengue sequelae	0.219 (0.1752–0.2628)	GBD (2019)^30^
RR_wolbachia	Relative risk of dengue incidence with the Wolbachia method	0.229 (0.1832–0.2748)	Utarini (2021)^6^
time_fatigue	Average duration of persistent syndrome (83.6 days) in years	0.233 (0.1864–0.2796)	Teixeira (2017)^31^
time_moderate	Average duration of moderate syndrome (7 days) in years	0.019 (0.0152–0.0228)	Xavier (2020)^32^
time_severe	Average duration of serious conditions (15 days) in years	0.041 (0.0328–0.0492)	Xavier (2020)^32^
Discount	Annual discount rate	0.050 (0.00–0.010)	Brazil (2014)^12^

ANS: National Supplementary Health Agency; CMED: Drug Market Regulation Chamber; BPS: Health Prices Database; ISPER: Public Employment and Income System; SIGTAP: Public Health Procedures Table; SIH/DATASUS: Hospital Information System; SINAN/DATASUS: Notifiable Diseases Information System; WMP: World Mosquito Program.

a Overall value for illustrative purpose, actual values adopting mean costs according to age range.

### Currency, price date, and conversion

All costs were valued in local currency (Brazilian Real – BRL) for 2022. When necessary, adjustments to inflation were made by adopting the Brazilian official inflation index (IPCA). The main results were presented in USD considering the yearly average exchange rate of USD 1:BRL 5 (http://www.ipeadata.gov.br).^23^

### Cost of dengue fever control program

A literature search was carried out to retrieve Brazilian studies on costs related to dengue adopting the keywords related to “dengue”; “costs” and “Brazil”. Two national articles were located for the Municipal Dengue Control Programs (PMCD): one for the city of Goiania^24^ and another for the city of São Paulo.^23^ Program costs were estimated considering human resources, surveillance and control actions, laboratory activities, communication (information and publicity) and related items detailed. A *per capita* value of BRL 6.02 was estimated for the city of São Paulo and BRL 35.01 for the city of Goiania. Assuming that the difference between the two values was related to their population density and their respective bargaining power, a regression model was proposed to integrate the values of the other simulated cities based on their total population.

### Cost of implementation of the Wolbachia method

The Wolbachia implementation costs have been obtained through the World Mosquito Program in Brazil, based on previous implementations in Rio de Janeiro, Niteroi, Belo Horizonte, Campo Grande and Petrolina and consider factors on the scale of implementation sites such as the number of inhabitants and geographic area sizes (Supplementary material). In addition to the direct costs of project activities during the preparation, release, and short-term monitoring phases, the estimated values include overhead costs incurred in Brazil (e.g., administration and management, laboratory and office facilities) and in WMP’s global program (e.g., digital and data systems, technical support, program oversight). The costs per inhabitant were estimated according to two implementation scenarios: a) municipality-led deployments using human resources from local public health departments to deliver Wolbachia mosquitoes or b) WMP-led deployments using WMP-employed human resources to deliver Wolbachia. Our base case model considered the first scenario with the mean per inhabitant cost of BRL 12.74 (CI 95%: 8.08–17.40). The second scenario was considered in a sensitivity analysis, with a mean per inhabitant cost of BRL 35.92 (CI95%: 29.12–42.72).

### Cost of hospitalization

The costs of hospital treatment for dengue in the Public Health System (SUS) in each municipality were collected from the Hospital Information System (SIH/SUS) records from 2008 to 2019. In addition, since the hospitalization values of the SIH/SUS are only related to reimbursement at the federal level, a correction factor of 3.51^33^^,^^34^ was adopted to consider the municipal and state expenses. These reimbursement values cover all medication and clinical interventions during each hospitalization.

For the private sector values, we considered the study of Machado and colleagues,^22^^,^^35^ who estimated direct and indirect costs in a prospective cohort of dengue cases in the city of Dourados, Brazil. Based on patients’ medical records, the average hospitalization cost BRL 2344.32.

### Cost of outpatient treatment

The cost of outpatient treatment was estimated from the Brazilian clinical guidelines.^36^ Based on the published recommendations, cost items were identified, quantified and valued in 2022 BRL. Each item was validated by an expert (infectious disease physician) and medication costs was collected from the National Health Price Database (BPS: http://bps.saude.gov.br/) for the public sector and from the Brazilian National Health Surveillance Agency (Anvisa) official price lists (CMED: https://www.gov.br/anvisa) for the private sector. Procedure costs were collected from the SUS Procedures Table Management System (SIGTAP: http://sigtap.datasus.gov.br/) in the public sector and Supplementary Health Regulatory Agency (ANS: https://www.gov.br/ans) in the private sector. Since the SIGTAP values are only related to reimbursement at the federal level, the same correction factor of 3.51 adopted in hospitalization reimbursements was considered.

### Cost of self-care treatment

The self-care treatment cost considered the use of antipyretics and analgesics for children and adults with mean dosages recommended by the MoH dengue guidelines.^36^ This cost was applied only to symptomatic patients and, thus, we considered that only the 12.5% of inapparent DENV infections who had symptoms (i.e., ‘self-care’ patients)^29^ would need medications for a median duration of 4 (IQR: 3–5) days.^37^

### Indirect costs for morbidity

The human capital theory was adopted to estimate the cost of premature death and disability due to dengue.^12^ In this analysis, data on formal income from the age of 18 until retirement age (62 years for women and 65 years for men) was considered. The mean income comprised the period from 2020 to 2021 and was based on the Information for the Public Employment and Income System (ISPER) database of the Ministry of Labour and Employment.^25^ A period of loss of productivity considered was 7 days (0.019 years) for outpatients and 11 days for hospitalized patients (0.041 years).^32^ For patients under 18 years old and elderly, indirect costs were assumed to cover the loss of productivity of a caregiver during the treatment period.

### Indirect costs for mortality

To calculate the costs related to lost productivity due to early mortality, the potential years of work lost (PYWL) for each gender and age at death were estimated considering a growth rate of labour income over time, based on an assumption that it would be equal to the average annual growth rate of Gross Domestic Product (GDP) in Brazil of 1.20% per year between 1970 and 2022 and a future income discount factor of 5%.

### Rationale and description of model

To consider both the epidemiologic dynamics of the incidence rate projections and the effect of clinical predictors (e.g., age and sex), an individual-based microsimulation State Transition Model (STM) structure was developed.^13^ This was particularly important to capture the specific community risk infection dynamics based on time, age and sex throughout the 20-year time horizon.

**Fig. 2:**
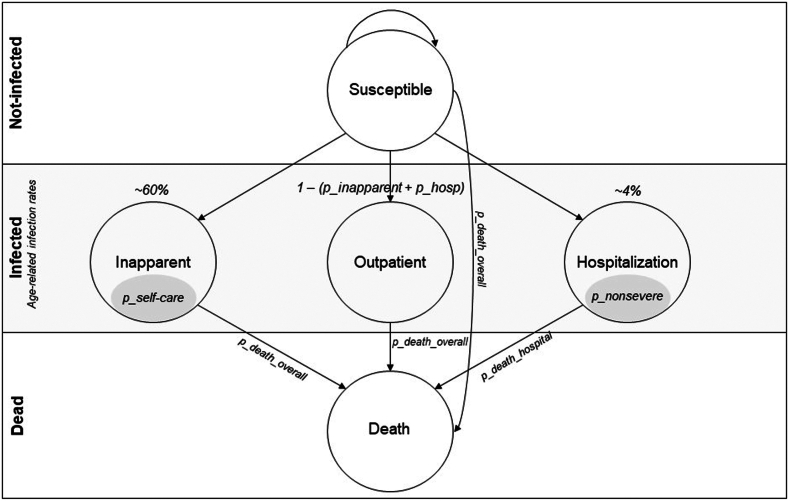
Health states and transitions in the dengue virus infection microsimulation model.

Our model structure compares the effectiveness of both dengue control strategies, as well as tracks the associated costs and clinical consequences in transitions between the different health states (Fig. 2):

a) Susceptible: Individuals who are vulnerable to dengue infection.
b) Inapparent Infection: This state accounts for individuals who have contracted the virus but show no apparent symptoms. It is further subdivided into two subgroups: asymptomatic inapparent (without symptoms) and inapparent with mild non-specific symptoms.
c) Outpatient Case: Individuals with more noticeable symptoms but not requiring hospitalization. Costs associated may include outpatient medical expenses, medications, and potential lost workdays.
d) Hospitalized Case: Individuals requiring hospitalization due to the severity of symptoms. Associated costs would encompass hospital expenses, specific treatments, and the duration of hospitalization.
e) Death: Represents fatal cases of the disease. Associated economic costs are related early death and the loss of productive life.

The model incorporates transitions between these states based on each city-specific dengue incidence rate data, the probability of hospitalization, and the mortality rate. In line with the dynamics of dengue infection, the model adopts the assumption that each individual can have only one yearly infection and a maximum of four infections during the entire time horizon. This is consistent with dengue immunity evidence, where following an infection with a specific serotype, an individual develops immunity against re-infection with that same serotype. Nonetheless, there is a potential for subsequent infection with a different serotype due to the transient nature of heterologous immunity, where cohort studies indicate that the protective immunity against different serotypes diminishes gradually within 1 or 2 years thereafter.^38^

### Infection probabilities

All projected infection probabilities were included in the model with a time-dependent framework, allowing for representation of the seasonality of the epidemics as well as immunity gained and lost from infections over time, expressed by the variation in the incidence of events over the years. In the alternative scenario, the effectiveness of the Wolbachia method was considered by reducing the infection probability by the protective efficacy, which was assumed to be equivalent for all four dengue virus serotypes.^6^ Given the unavailability of a long-term experimental study of Wolbachia, our simulation considers its estimates of effectiveness on a proposed counterfactual method, assuming two proposed scenarios, one with and one without the implementation of the Wolbachia method in each of the cities.^39^

The reference dengue incidence rate (without Wolbachia) and its age distribution in cities were derived from suspected cases reported to SINAN. Using data from 2001 to 2019 and city demographics, we projected future incidence from 2023 to simulate city-specific dengue patterns. This included analyzing the last decade’s incidence cycles and peaks to forecast future risks. This approach replicates the dengue risk pattern observed in the previous 10 years, following model face validation recommendations. Nevertheless, even if adopting the same trends (here understood as the risk patterns), the number of cases and their respective distribution by age group would not be the same as in the last 10 years, as it incorporates the demographic changes each city has undergone. In Niteroi, Wolbachia-infected mosquito releases began in 2017, significantly reducing dengue incidence due to 80% city coverage.^8^ To counteract information bias, Niteroi’s projections started from 2016, not 2023. Additionally, risk projections using multiple regression models on time series were developed and validated, assessing the best models by visual inspection and Mean Absolute Percentage Error (MAPE). However, due to the long-time horizon, these models couldn’t surpass the initial replication strategy (Supplementary material).

To take into account the under-reporting of dengue cases to the national surveillance system in parameterizing the economic model, an expansion factor of 12 was applied to the dengue incidence estimates derived from SINAN, consistent with the mean expansion factor of 12.3 (IC95%: 6.7–20.8) suggested by the Global Burden of Disease (GBD) Collaboration.^40^ The effectiveness of Wolbachia deployments in reducing dengue incidence was assumed to be 77.1%, based on the protective efficacy measured in an Indonesian cluster-randomized trial.^6^ This risk reduction was applied to the projected incidence for each of the seven cities. A summary of this and other parameters of the model can be found in Table 1.

### Disease severity, hospitalization, and lethality

The inapparent proportion among all dengue infections (approximately 60%) was informed by a study from Brazil,^27^ which was broadly consistent with a recent study in Iquitos, Peru,^28^ that reported an inapparent:symptomatic ratio of 1.4:1 (i.e., 58.3% inapparent) among dengue infections detected in a prospective community-based study, with inapparent infections including individuals who were either fully asymptomatic or had undetermined DENV symptoms.

The hospitalization rate was based on real-world data from each of the 7 cities considered. According to SINAN data, the hospitalization rate of suspected dengue cases between 2008 and 2019 ranged from 0.83% to 8.92%, depending on individual age. Each rate was converted to a hospitalization probability according to the exponential method. Based on the SIH data, the proportion of severe dengue cases ranged from 3.91% to 26.29% and the Case Fatality Rate (CFR) ranged from 0.16% to 7.75% depending on individual age.

### Main assumptions

The main assumptions of the model can be summarized as: a) Each individual can have a single infection per year. Although being possible to have more infections in a year, this is not common and was not included in the reference case. The total number of infections along the time horizon was limited to four, regardless of the number of possible serotypes available; b) The average risk of infection is applied by each age range; c) Consistent with real-world estimates, we have assumed that all hospitalized cases were severe; d) Lethality occurs only in hospitalized cases. Other cases can die with the same risk as the general population, following the Brazilian population’s general mortality; d) The Wolbachia method has no impact on current program resources. Potential future savings to the dengue control program in the case of lower dengue incidence are still uncertain and thus not included in the model. It is also important to highlight that, consistent with the available effectiveness data, our model assumes as minimum coverage a Wolbachia prevalence in *Ae. aegypti* above 60%.^9^

### Characterizing heterogeneity and uncertainty

Local heterogeneity was considered by presenting model results for each simulated city, as the underlying data consider the age and sex distribution of each city and their relationship with life expectancy, average wage, and consequent productivity loss impact, as well as the particular age and sex risk of being infected. The number of first order Monte Carlo iterations were based on model convergence, being consistent with the population size of each city: Sao Paulo (6 million iterations), Belo Horizonte (3 million iterations), Campo Grande (3 million iterations), Fortaleza (1 million iterations), Goiania (3 million iterations), Manaus (2 million iterations) and Niteroi (200 thousand iterations).

In addition to the first-order Monte Carlo simulations, the parameter uncertainty was also handled through deterministic sensitivity analysis (Tornado) covering the main parameter values and their ranges (Table 1) and probabilistic sensitivity analysis (PSA) through 10,000 s-order Monte Carlo simulations for each city.^14^ When applicable, the range values were based on the imprecision estimate (95% confidence interval limits) or a minimum range of ±20% of the point estimate. Structural and methodological uncertainty were also considered with a series of alternative model versions (scenarios) of different perspectives (Society, Health sector and Public Health system), time horizon (5, 10 and 20 years) and implementation methods (municipalities and WMP team).

### Engagement and validation

A face validation of the model was conducted through a meeting with representatives from the Ministry of Health and other selected stakeholders (public health managers) in December 2022. Feedback and suggestions were included in the revised model.

### Role of the funding source

This work was funded by the Wellcome Trust under a grant (224459/Z/21/Z) to the World Mosquito Program (WMP). The funding source had no role in the design of this study and its execution, analyses, or decision to submit results.

## Results

### Base case

Based on each age and sex risk projection, the microsimulation transition model was able to incorporate the dynamics of the incidence rate profile for each of the seven cities (Supplementary Fig. S3). In the absence of Wolbachia, the model projected a total of 1,762,688 dengue cases (excluding inapparent infections) throughout the 20-year time horizon (from 2023 to 2042). With the implementation of Wolbachia, it would be possible to avert a total of 1,295,566 dengue cases during this period (Table 2). In all seven cities, there were total cost differences favouring the use of the Wolbachia method over the 5, 10 and 20-year horizon. The average total cost difference per 1000 people exceeded values of BRL 2.5 billion, and for individual cities, absolute values ranged from - USD 99,658.31 (-BRL 498,291.54) in Manaus, to – USD 1,171,060.49 (- BRL 5,855,302.49) in Goiania. The main factor contributing to this cost difference was the indirect costs averted. When only direct costs were considered, the cost difference was lower and in the two cities with lower dengue burden, there was a net positive cost difference: Manaus, with USD 97.90 (BRL 489.51) and Sao Paulo, with USD 1253.08 (BRL 6265.40). Thus, for five of seven cities, the Wolbachia intervention was predicted to generate net cost savings even considering only the direct costs averted (i.e., from a healthcare perspective).

**Table 2:** Costs and benefits of *Wolbachia* deployments in seven Brazilian cities over 5-, 10- and 20-year horizon.

City	Costs (per 1000 people)		Cost difference (per 1000 people)	Dengue cases^a^		Dengue cases averted	DALY averted (per 1000 people)	ICER	NMB
	Without Wolbachia	With Wolbachia		Without Wolbachia	With Wolbachia				
**5 years horizon**									
Manaus	R$ 9,321,291.94	R$ 9,086,340.22	-R$ 234,951.71	27,107	6430	20,677	0.53	Dominance	R$ 298.61
São Paulo	R$ 11,674,360.94	R$ 11,401,176.00	-R$ 273,184.94	103,141	24,638	78,503	1.78	Dominance	R$ 486.42
Fortaleza	R$ 8,499,011.86	R$ 8,007,715.25	-R$ 491,296.61	79,985	19,781	60,204	1.33	Dominance	R$ 651.36
Niteroi	R$ 9,295,178.36	R$ 8,324,805.54	-R$ 970,372.82	21,403	5141	16,262	7.03	Dominance	R$ 1813.86
Belo Horizonte	R$ 11,131,092.17	R$ 10,717,806.68	-R$ 413,285.49	195,659	47,966	147,692	1.75	Dominance	R$ 622.70
Campo Grande	R$ 12,528,204.37	R$ 10,896,787.72	-R$ 1,631,416.65	60,574	14,836	45,738	2.17	Dominance	R$ 1892.11
Goiania	R$ 11,674,360.94	R$ 11,401,176.00	-R$ 273,184.94	167,060	46,942	120,119	6.66	Dominance	R$ 1072.11
**Overall (mean)**	**R$ 10,589,071.51**	**R$ 9,976,543.92**	**-R$ 612,527.59**	**93,561**	**23,676**	**69,885**	**3.04**	**Dominance**	**R$ 976.74**
**10 years horizon**									
Manaus	R$ 18,321,062.87	R$ 18,017,601.69	-R$ 303,461.19	36,492	8643	27,849	0.62	Dominance	R$ 377.26
São Paulo	R$ 22,621,130.00	R$ 22,290,670.00	-R$ 330,460.00	136,618	32,550	104,068	2.00	Dominance	R$ 570.46
Fortaleza	R$ 16,553,320.00	R$ 15,750,780.00	-R$ 802,540.00	138,690	34,102	104,587	3.00	Dominance	R$ 1162.54
Niteroi	R$ 17,344,900.00	R$ 15,877,620.00	-R$ 1,467,280.00	36,273	8924	27,350	10.00	Dominance	R$ 2667.28
Belo Horizonte	R$ 25,145,810.00	R$ 22,258,670.00	-R$ 2,887,140.00	286,541	69,796	216,745	2.50	Dominance	R$ 3187.14
Campo Grande	R$ 20,438,936.74	R$ 19,884,587.22	-R$ 554,349.52	101,032	25,270	75,762	5.76	Dominance	R$ 1245.33
Goiania	R$ 25,256,470.00	R$ 21,684,930.00	-R$ 3,571,540.00	243,356	67,451	175,904	8.40	Dominance	R$ 4579.54
**Overall (mean)**	**R$ 20,811,661.37**	**R$ 19,394,979.84**	**-R$ 1,416,681.53**	**139,857**	**35,248**	**104,609**	**4.61**	**Dominance**	**R$ 1969.94**
**20 years horizon**									
Manaus	R$ 35,757,671.08	R$ 35,259,379.55	-R$ 498,291.54	69,081	16,406	52,675	0.46	Dominance	R$ 553.59
São Paulo	R$ 42,719,776.61	R$ 42,113,747.62	-R$ 606,028.99	264,432	62,923	201,509	4.47	Dominance	R$ 1142.53
Fortaleza	R$ 31,782,689.66	R$ 30,361,540.46	-R$ 1,421,149.19	257,115	63,662	193,453	9.44	Dominance	R$ 2554.02
Niteroi	R$ 40,937,200.06	R$ 39,290,941.43	-R$ 1,646,258.62	64,194	16,170	48,024	13.57	Dominance	R$ 3274.53
Belo Horizonte	R$ 46,235,135.08	R$ 41,585,954.48	-R$ 4,649,180.59	515,368	130,743	384,625	3.34	Dominance	R$ 5050.53
Campo Grande	R$ 44,470,163.36	R$ 40,308,195.96	-R$ 4,161,967.40	184,245	47,971	136,274	8.62	Dominance	R$ 5196.63
Goiania	R$ 46,928,290.54	R$ 41,072,988.05	-R$ 5,855,302.49	408,253	129,247	279,006	9.51	Dominance	R$ 6995.95
**Overall (mean)**	**R$ 41,261,560.91**	**R$ 38,570,392.51**	**-R$ 2,691,168.40**	**251,813**	**66,732**	**185,081**	**7.06**	**Dominance**	**R$ 3538.25**

DALY: Disability-adjusted life years; ICER: Incremental cost-effectiveness ratio; NMB: Net monetary benefit (incremental).

a Reported cases: excluding inapparent infections (asymptomatic and self-care cases).

The Wolbachia intervention was projected to avert between 0.46 (Manaus) and 9.51 (Goiania) DALYs per 1000 inhabitants over 20 years (Table 2). It was estimated that the addition of Wolbachia to the current dengue control strategies would be associated with an expected outcome of absolute dominance (that is, lower cost and greater effectiveness) in all seven simulated cities.

Adopting the official BRL 120 thousand per DALY cost-effectiveness threshold for neglected diseases, the incremental Net Monetary benefits (NMB) per inhabitant was positive in all seven cities, ranging from USD 110.72 (BRL 553.59), in Manaus, to USD 1399.19 (BRL 6995.95), in Goiania. The incremental benefit-cost ratio (BCR) indicates that each additional BRL per inhabitant invested for the implementation of the Wolbachia method was associated with a positive return of USD 707.65 (BRL 3538.25) on average, and ranging from USD 8.89 (BRL 44.45) in Manaus to USD 110.02 (BRL 550.13) in Goiania, indicating that the implementation of Wolbachia is likely to generate substantial net economic benefits in the Brazilian context (Table 2).

### Sensitivity analysis

**Fig. 3:**
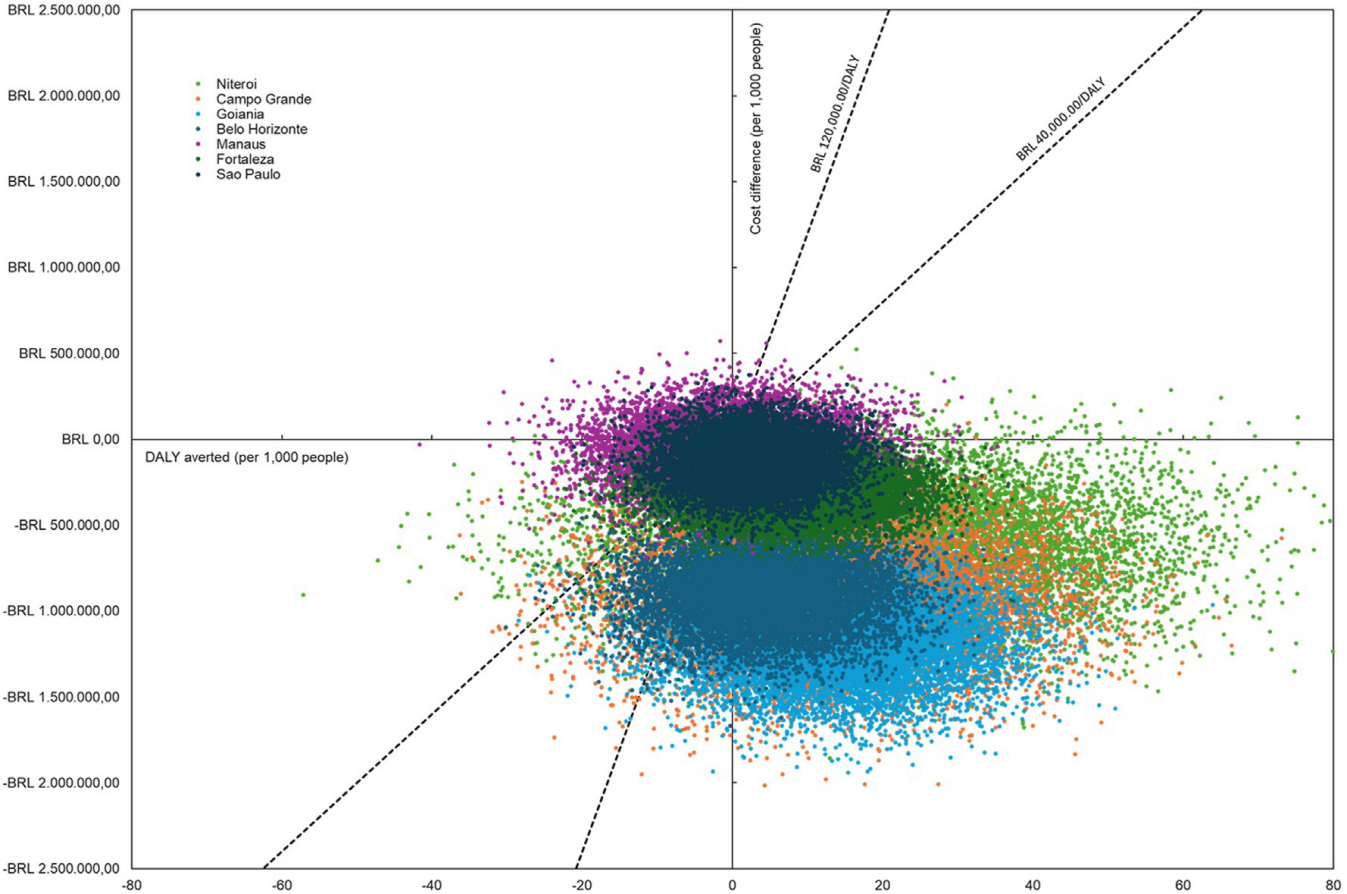
Cost-effectiveness plane of the implementation of the Wolbachia method compared to the common dengue control program strategies in Brazilian cities from 2023 to 2042 (10,000 Monte Carlo simulations). *Note:* Dashed lines reflect both reference (BRL 40,000.00/DALY) and neglected diseases (BRL 120,000.00/DALY) cost-effectiveness thresholds in Brazil.

Based on the global sensitivity analysis of the uncertainty, the Tornado diagram indicated the expansion factor and Wolbachia effectiveness (Relative Risk) as the main uncertainty factors. Nevertheless, although affected, the incremental NMB stayed positive in all variations. After sequential stochastic trials (1st order Monte Carlo) of the base case scenario, it was possible to expect a convergence on the global expected value of incremental NMB around USD 290 (BRL 1450.00), also supporting the robustness of the model conclusions in favour of the cost-effectiveness of the Wolbachia method. As illustrated in Fig. 3, most of the cities had more than 95% of the simulations below the cost-effectiveness thresholds of BRL 40 thousand (reference) and BRL 120 thousand (neglected diseases) per DALY used in Brazil, as well as situated inside the lower-right quadrant of the cost-effectiveness plane (i.e., cost-saving), hence concluding that the addition of Wolbachia is probably a cost-effective strategy in the Brazilian context. Only São Paulo and Manaus had less favourable scenarios, with 80% and 56% of the simulations below both thresholds, respectively.

### Scenario analysis

In shorter time horizons of 5 or 10 years, the incremental NMB still showed the *Wolbachia* method to be a cost-effective option compared to the common dengue control strategy, although the magnitude of the NMB was lower when compared to the 20-year time horizon. In addition, although absolute dominance was not maintained for all the seven cities in the Health sector and Public Health system perspectives, the ICERs estimated for Manaus and São Paulo remained far below the Brazilian cost-effectiveness threshold of BRL 120 thousand, with NMB still positive in both scenarios for all the cities (Supplementary material). Even considering an average implementation cost more than two times higher based on the implementation of the Wolbachia method by the WMP team instead of the municipalities, the Wolbachia method was shown to be a dominant and cost-effective strategy with a positive incremental NMB in all seven cities simulated. Alternative scenarios with variations in the discount rate also did not significantly alter the conclusion about the cost-effectiveness of the Wolbachia method.

## Discussion

Wolbachia has been used in several countries as a tool to control dengue and other Aedes-borne diseases by either suppressing (with releases of males only) or replacing (with releases of males and females) *Ae. aegypti* mosquito populations. Herein, we have modelled the health and economic impacts of implementing the Wolbachia replacement strategy,^41^ used by the World Mosquito Program, at a large scale in Brazil. In all the seven Brazilian cities simulated, Wolbachia release was associated with a reduction in costs due to fewer hospitalizations and productivity loss, as well as clinical benefits in terms of DALYs averted. For every 1000 inhabitants, there was a projected cost difference of up to USD 1,171,060.50 (BRL 5,855,302.49) and a total of 9.5 DALYs averted in favour of the Wolbachia method over a 20-year horizon. On average, each BRL invested in the implementation of the Wolbachia method was projected to return BRL 278.73 in economic benefits, with this return on investment ranging from BRL 44.45 to BRL 550.13 for individual cities.

In addition to the difference in baseline dengue burden, other sociodemographic differences that may explain the variation in cost differences and ICERs between the simulated cities, include the age structure, the income of the economically active population, and the cost of healthcare in each region analyzed. Both Niteroi and Fortaleza had twice the rate of hospitalised cases compared to Belo Horizonte, and Niteroi in particular had a higher proportion of severe dengue cases, which incur a higher DALY burden than uncomplicated cases. Also, Fortaleza and Niteroi had almost 3 times the CFR when compared to Belo Horizonte. These differences in the assumed severity distribution of dengue cases among the cities, which were derived from data from official health information systems in each city, can explain why the DALY burden averted is not necessarily proportionate to the number of dengue cases averted.

Our cost-effectiveness findings were consistent with previous economic evaluations in local and international contexts. Based on dengue incidence retrospective, Soh and colleagues^42^ have estimated that a national Wolbachia suppression program in Singapore would lead to an estimated $329.40 Million saved in economic costs from 2010 to 2020 even under a 40% intervention efficacy. In Thailand, although not formally compared, Wolbachia was shown to be cost-effective ($343 per DALY averted) vs. other single control measures in exploratory analyses also conducted by Knerer and colleagues.^43^ In Brazil, a recent study by Barbosa and colleagues,^44^ restricted to the public health system perspective of the state of Goias, also found that adding the implementation of wMel Wolbachia to the conventional vector control program would be a cost-effective strategy, with an incremental cost of BRL 72,200 per QALY gained.

Our study has a series of strengths that we should highlight. Adopting the microsimulation model and the societal perspective, we were able to reflect the effect of individual and local characteristics on costs and benefits in each of the seven cities. The number of projected dengue cases in the absence of Wolbachia was very consistent with the total of 1,911,084 cases reported to the Ministry of Health during a previous 20-year period (from 2002 to 2021) in the same cities, suggesting that the model projections were very robust. All the parameters were included in sensitivity analysis, and, despite some important impacts on the model outcomes, the conclusions were consistently in favour of the implementation of the Wolbachia method. The same situation was observed when running a series of different scenario analyses. None of the different scenarios tested changed the conclusion on the cost-effectiveness of the Wolbachia method compared to the current dengue control strategy. Even under scenarios that resulted in a net positive incremental cost of the intervention in some cities and time frames, the intervention was still found to be cost-effective. These results are consistent with previous international studies in Indonesia^45^ and Vietnam,^46^ where the Wolbachia method was also found to be cost-effective. Considering the cost-effectiveness criteria adopted by the Brazilian National Commission for the Incorporation of Technology in the SUS (CONITEC) for neglected diseases (BRL 120 thousand per DALY), the results of the Wolbachia method would fall far below this threshold.

### Limitations

Some limitations of our study should be considered when interpreting our results. First, the Wolbachia effectiveness estimates in our model consider a >60% prevalence of the wMel strain. However, studies with lower coverages and different strains achieved smaller reductions, around 40%, in dengue cases.^9^^,^^47^ We assumed that the introduction of the Wolbachia method would have no impact on current program resources. Although the projected reductions in dengue incidence are likely to result in potential future savings for dengue control programs, our estimates for these savings would be extremely uncertain. Not including this benefit in the model was a very conservative decision. Our results were designed to reflect seven specific Brazilian cities. Because of differences related to dengue burden and demographics, we would expect variability in the results for other cities not included in our model. Adopting the city of notification instead of the city of residence could be an issue in the true geographical distribution of the disease in the population when the notification location differs from the residence. This discrepancy can arise due to displacements, travel, or accessing healthcare services in different localities. Nevertheless, our strategy seeks to minimize the impacts of underreporting commonly found in health databases. In addition, the case expansion factor may also be acknowledged as a limitation, its adoption remains consistent with challenges observed in notification systems and real-world data, emphasizing the significance of underreporting of inapparent cases. Given the complexities and limitations in the reporting infrastructure, the use of a case expansion factor allows for a more comprehensive reflection of the true disease burden, accounting for cases that may go unnoticed or unreported. In addition, the value of the expansion factor was consistent with previous literature, highlighting the robust research conducted by Silva et al.,^26^ who, based on active searches in the community, identified the actual prevalence of dengue cases. With access to notifications registered in SINAN, the authors conclude that there were 12 dengue cases per reported case in the community, similar to the values adopted in our model.

Collectively, our results suggest that the implementation of the Wolbachia method is a cost-effective strategy for dengue control, presenting an absolute dominance to the current dengue control strategy in seven different Brazilian cities.

## Contributors

IRZ, RRAF, MGSC, MP and HMP contributed to the conception and design of the study. IRZ, MGSC, MP and HMP were involved in data collection. IRZ and RRAF directly accessed, verified the underlying data and performed the data analysis. IRZ was responsible for the decision to submit the manuscript. All authors contributed to the interpretation of the results, writing of the manuscript, critical review, and approval of the final text.

## Data sharing statement

The data and model files can be shared for non-commercial purposes with those who provide a methodologically sound proposal. Proposals should be directed to the corresponding author; to gain access, requestors will need to sign a data access agreement.

## Editor note

The Lancet Group takes a neutral position with respect to territorial claims in published maps and institutional affiliations.

## Declaration of interests

None declared.

## Appendix A Supplementary data

Supplementary Figs. S1–S4 and Tables S1–S12

Translated_Summary_Portugues
