# Supplementary material for: Simulation-based economic evaluation of the Wolbachia method in Brazil: a cost-effective strategy for dengue control
Source: Lancet Reg Health Am. 2024 Jun 3;35:100783. doi: 10.1016/j.lana.2024.100783 (PMC11190723; doi:10.1016/j.lana.2024.100783)
Supplement: Supplementary Figs. S1–S4 and Tables S1–S12 [file mmc1.pdf]

Supplementary Material

Figures

Figure S 1. Observed and projected incidence rate of suspected cases of Dengue according to age range during 2001 to 2042 in: A) Manaus, AM; B) Fortaleza, CE; C) Belo Horizonte, MG; D) Niterói, RJ; E) São Paulo, SP; F) Campo Grande, MS and G) Goiânia, GO. Source: Based on data from Datasus, Ministry of Health. 2
Figure S 2. Number of cases of dengue observed (n=156, 2001/1 to 2013/12) and predicted (n=36, 2014/1 to 2016/12) in the municipality of Niteroi according to regression models (ETS, HW, ARIMA and TBATS models). ... 3
Figure S 3. Incidence rate of suspected cases of Dengue according to age range during 2001 to 2021 in: A) Manaus, AM; B) Fortaleza, CE; C) Belo Horizonte, MG; D) Niterói, RJ; E) São Paulo, SP; F) Campo Grande, MS and G) Goiânia, GO. Source: Datasus, Ministry of Health. 4
Figure S 4. Tornado diagram on incremental Net Monetary Benefit (NMB) of the implementation of Wolbachia method compared to the current dengue control program strategies in Brazilian cities during 2023 to 2042. 5

Tables

Table S 1. Age and sex distribution of the population included in the complete model, according to the year 2021. . 6
Table S 2. Mean public reimbursement (BRL) of dengue hospitalizations according to severity in Brazilian cities from 2016 to 2019. 7
Table S 3. Cost of outpatient treatment of dengue cases (BRL). 8
Table **S 4**. Cost of symptomatic inapparent dengue case treatment (self-care). 9
Table S 5. Productivity loss costs (BRL) due to outpatient period among patients with dengue, categorized by age range and gender in Brazilian cities. 10
Table S 6. Productivity loss costs (BRL) due to hospitalization period among patients with dengue, categorized by age range and gender in Brazilian cities. 11
Table S 7. Productivity loss costs (BRL) due to premature mortality among patients with dengue, categorized by age range and gender in Brazilian cities. 12
Table S 8. Hospitalization rate of suspected dengue cases in Brazilian cities during 2008 to 2019. 14
Table S 9. Severe rates of dengue hospitalizations in Brazilian cities during 2016 to 2019. 14
Table S 10. Case fatality rate of dengue hospitalizations in Brazilian cities during 2016 to 2019. 14
Table S 11. Direct and indirect costs according to the seven cities during the 20 years horizon 15
Table S 12. Scenario analysis considering the Health sector and Public Health system perspective and the implementation strategy of Wolbachia method compared to the common dengue control program strategies in Brazilian cities during 2023 to 2042. 16

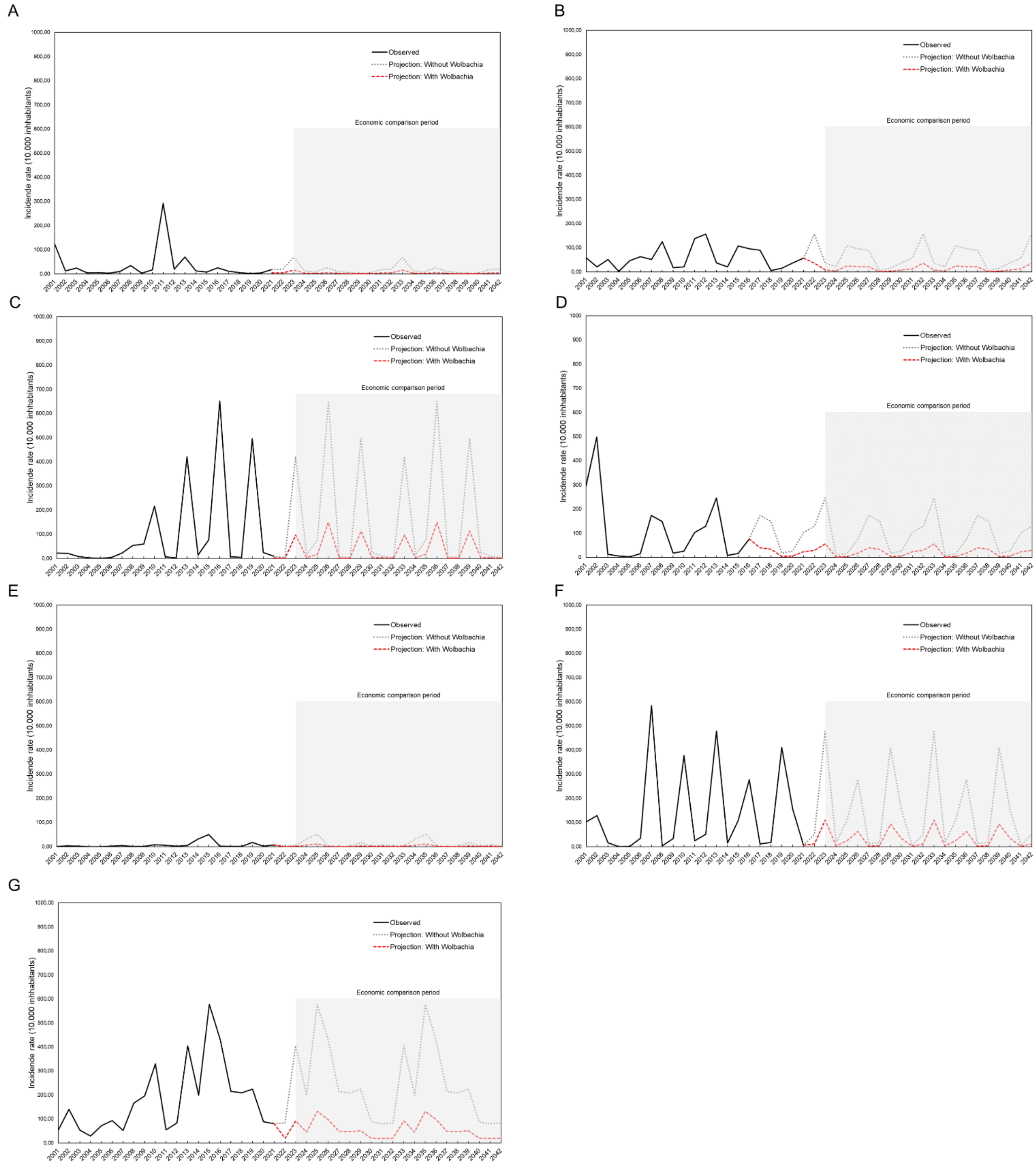

Figure S 1. Observed and projected incidence rate of suspected cases of Dengue according to age range during 2001 to 2042 in: A) Manaus, AM; B) Fortaleza, CE; C) Belo Horizonte, MG; D) Niterói, RJ; E) São Paulo, SP; F) Campo Grande, MS and G) Goiânia, GO. Source: Based on data from Datasus, Ministry of Health.

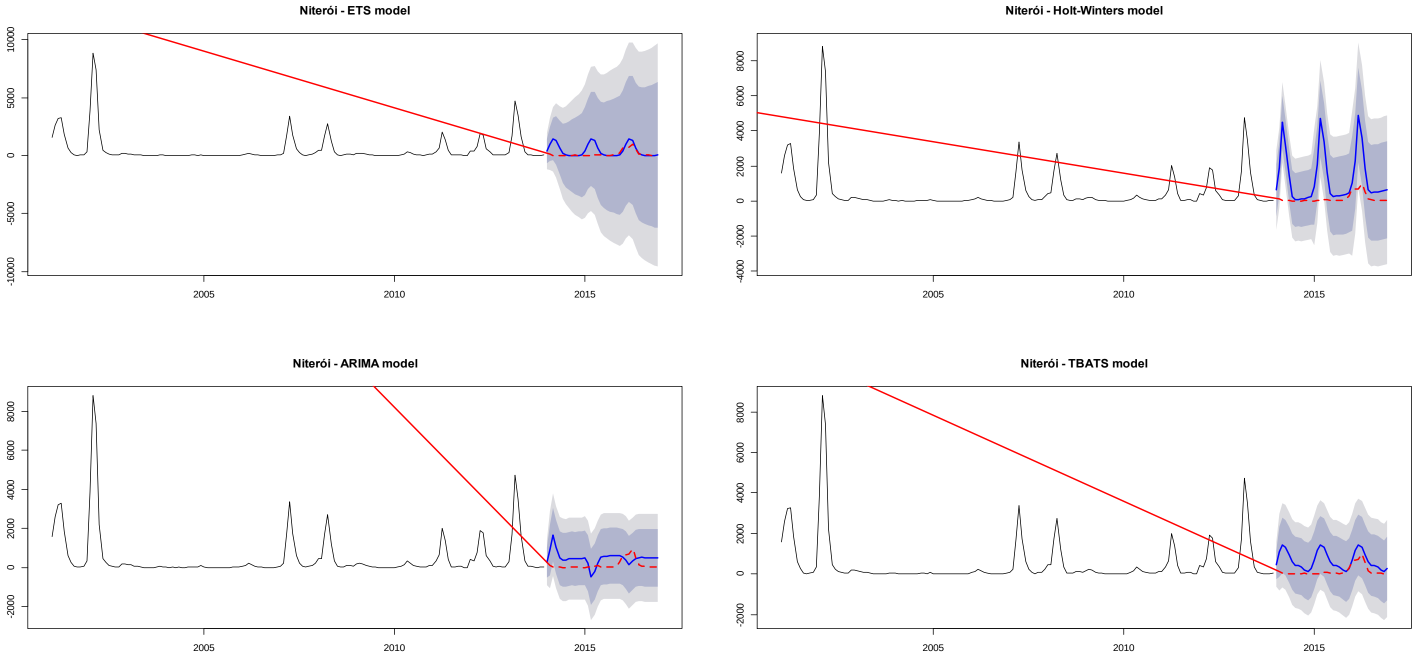

Figure S 2. Number of cases of dengue observed (n=156, 2001/1 to 2013/12) and predicted (n=36, 2014/1 to 2016/12) in the municipality of Niteroi according to regression models (ETS, HW, ARIMA and TBATS models).

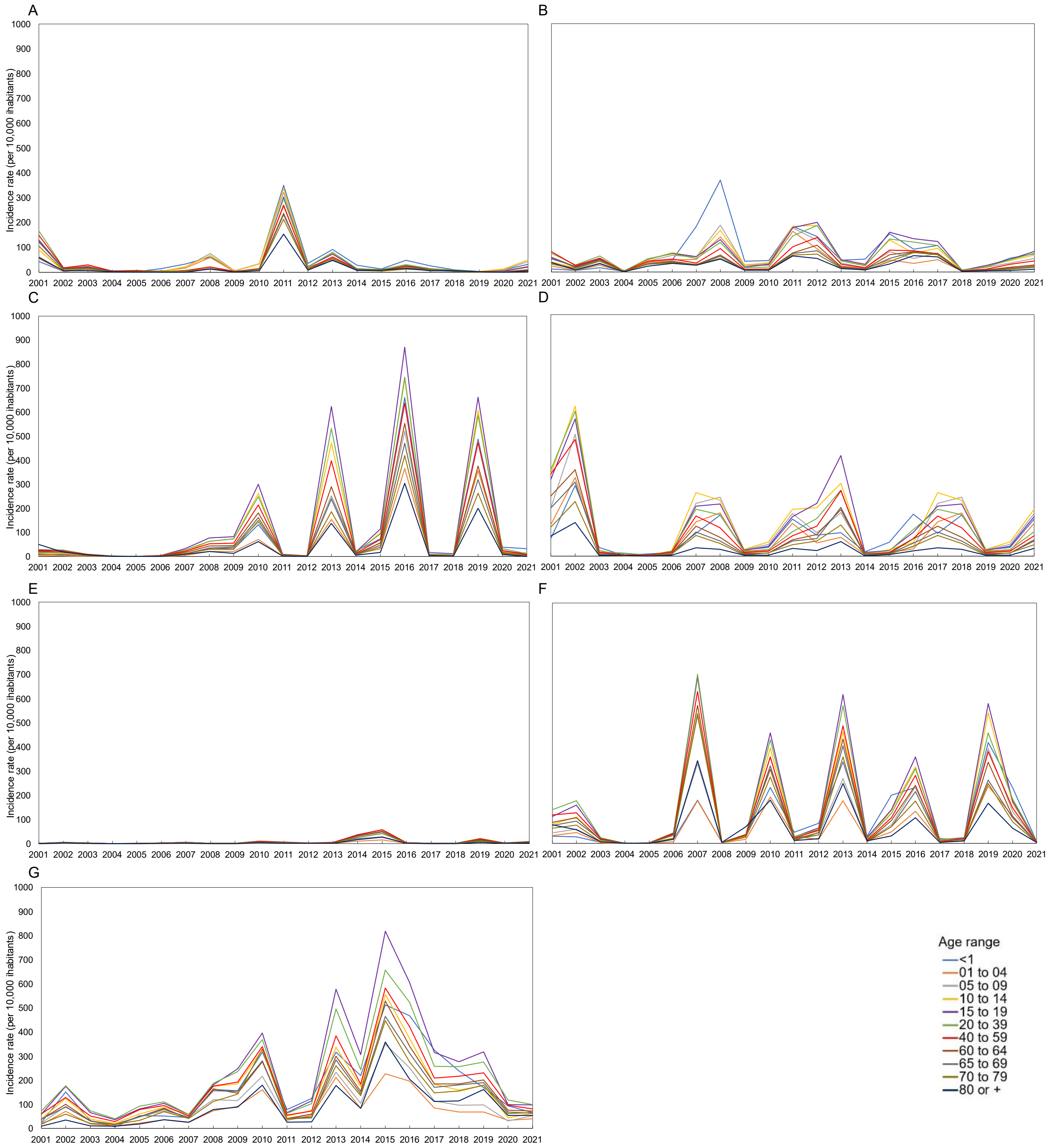

Figure S 3. Incidence rate of suspected cases of Dengue according to age range during 2001 to 2021 in: A) Manaus, AM; B) Fortaleza, CE; C) Belo Horizonte, MG; D) Niterói, RJ; E) São Paulo, SP; F) Campo Grande, MS and G) Goiânia, GO. Source: Datasus, Ministry of Health.

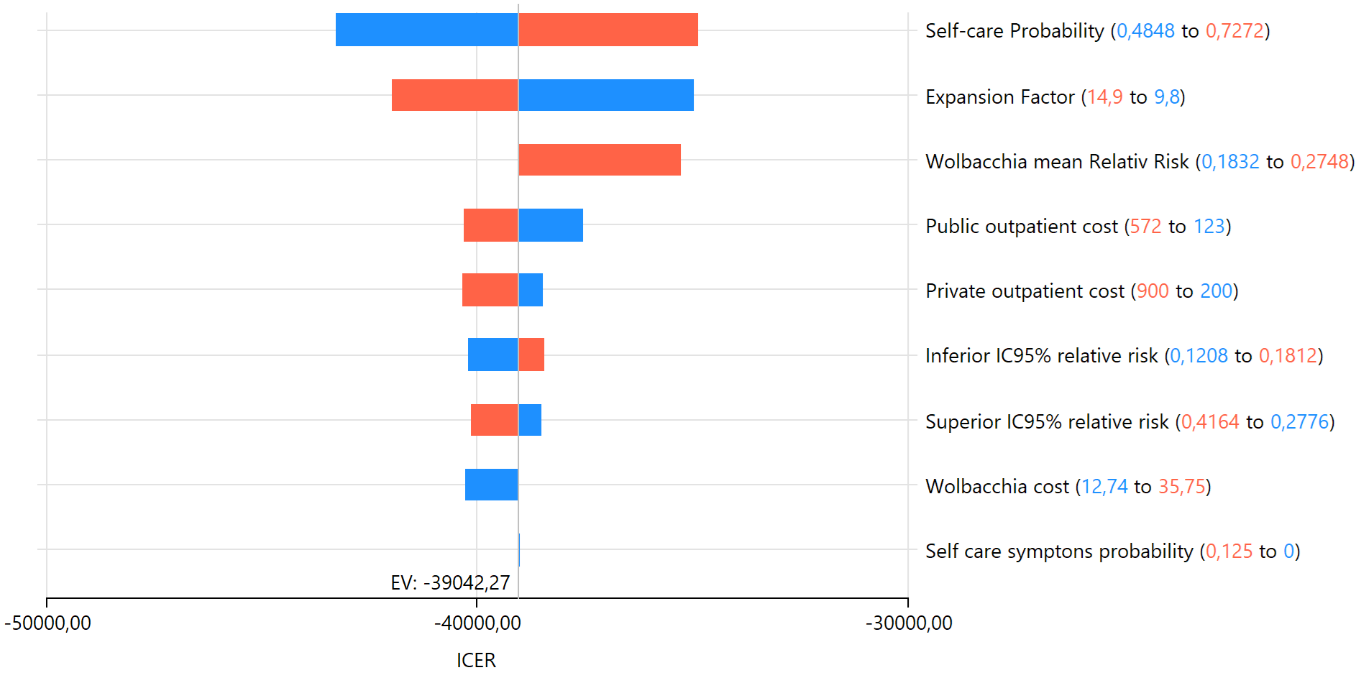

Figure S 4. Tornado diagram on incremental Net Monetary Benefit (NMB) of the implementation of Wolbachia method compared to the current dengue control program strategies in Brazilian cities during 2023 to 2042.

Table S 1. Age and sex distribution of the population included in the complete model, according to the year 2021.

Age range (years)	Niterói		São Paulo		Belo Horizonte		Goiânia		Campo Grande		Fortaleza		Manaus	
	**Male**	**Total**	**Male**	**Total**	**Male**	**Total**	**Male**	**Total**	**Male**	**Total**	**Male**	**Total**	**Male**	**Total**
0 to 4	12,910	25,222	399,006	779,817	66,712	130,323	48,347	94,615	32,289	63,135	83,959	164,077	88,513	173,176
5 to 9	13,033	25,507	401,526	787,114	67,111	131,712	51,367	101,124	32,893	64,372	83,972	165,583	92,425	182,624
10 to 14	13,099	25,790	395,062	779,315	72,134	143,060	53,522	106,411	32,629	63,802	94,571	188,701	98,113	195,468
15 to 19	14,468	28,477	424,243	841,885	84,841	169,260	55,304	110,727	33,000	64,537	105,638	211,469	100,622	199,775
20 to 29	33,588	66,955	915,969	1,832,490	187,151	376,127	116,639	234,705	70,838	140,350	225,605	455,865	204,178	413,206
30 to 39	38,394	79,247	983,123	2,015,873	196,537	411,541	134,376	276,889	73,154	149,247	219,641	468,676	182,562	378,413
40 to 49	35,779	76,159	867,619	1,845,307	177,680	381,835	112,492	237,048	61,523	130,383	173,946	383,999	148,408	313,557
50 to 59	30,786	67,823	670,346	1,463,257	140,202	313,025	80,184	177,172	48,127	105,443	134,623	307,616	97,538	204,616
60 to 69	26,099	61,476	476,557	1,110,842	106,853	252,485	52,989	124,842	33,130	75,882	81,517	200,003	54,559	120,925
70 to 79	14,794	38,085	245,895	618,561	57,842	145,185	26,004	64,361	16,446	39,887	41,052	107,909	22,298	52,956
80 or more	6,960	22,240	106,029	321,911	25,881	76,148	10,004	27,732	7,093	18,963	15,190	49,493	7,399	21,187
**Total**	239,910	516,981	5,885,375	12,396,372	1,182,944	2,530,701	741,228	1,555,626	441,122	916,001	1,259,714	2,703,391	1,096,615	2,255,903

Source: Department of Health Informatics, Ministry of Health.

Table S 2. Mean public reimbursement (BRL) of dengue hospitalizations according to severity in Brazilian cities from 2016 to 2019.

Age range (years)	Niterói		São Paulo		Belo Horizonte		Goiânia		Campo Grande		Fortaleza		Manaus	
	**Moderate**	**Severe**	**Moderate**	**Severe**	**Moderate**	**Severe**	**Moderate**	**Severe**	**Moderate**	**Severe**	**Moderate**	**Severe**	**Moderate**	**Severe**
00 to 19	1161·76	2225·6	1316·21	3047·24	1428·79	3856·69	1059·74	2778·99	1161·37	2355·75	1152·42	2581·22	1154·44	2392·38
20 to 39	1191·16	2395·63	1334·42	4190·47	1468·41	3677·54	1162·01	3416·02	1549·69	3475·03	1148·76	2750·9	1230·33	3132·52
40 to 59	1161·63	2437·42	1300·43	4336·6	1491·7	3501·00	1107·55	2805·66	1229·76	2452·24	1185·53	3220·48	1261·64	2508·18
60 to 79	1223·78	2486·46	1504·05	3033·02	1537·91	4465·5	1303·7	3386·26	1506·77	2932·42	1497·22	5278·73	1106·1	2358·46
80 or more	1702·85	2963·36	1568·79	11854·15	1872·21	3868·11	1381·7	4391·94	1342·6	5544·97	1549·98	3858·8	1120·42	2138·29
Overall	1288·24	2501·69	1404·78	5292·29	1559·8	3873·77	1202·94	3355·78	1358·04	3352·08	1306·78	3538·03	1174·59	2505·97

Source: Datasus, Ministry of Health.

Table S 3. Cost of outpatient treatment of dengue cases (BRL).

Item	Quantity	Public		Private		Source
		Unit cost	Total Cost	Unit cost	Total Cost	
Medical visit	2	35·10	70·20	93·04	186·08	SIGTAP; ANS
Total blood count	2	14·43	28·85	8·99	17·98	SIGTAP; ANS
Dengue test	1	263·25	263·25	185·13	185·13	SIGTAP; ANS
Dipyrone 500mg/ml (2 mL)	1	1·46	1·46	1·73	1·73	BPS; CMED
Syringe with needle (3 mL)	1	0·37	0·37	3·46	3·46	BPS; CMED
Oral rehydration salts	4	0·72	2·88	5·00	20·00	BPS; CMED
**Total**			**367.01**		**414.39**	

Note: SIGTAP: SUS Procedures Table Management System; ANS: Supplementary Health Regulatory Agency; BPS: National Health Price Database; CMED: Drug market regulation chamber council.

Table **S 4**. Cost of symptomatic inapparent dengue case treatment (self-care).

**Population**	**Item**	**Daily units (mg)**	**Symptoms duration (days)**	**Total units (mg)**	**Costs (per tablet or mL)**	**Total costs**	**Source**
Adult	Dipyrone tablet<br>(500 mg tablet every 6 hours)1	2000	4	8000	R$ 0·93	R$ 14·93	CMED
Children	Dipyrone oral solution<br>(10 mg/kg every 6 hours)2	10003	4	4000	R$ 2·66	R$ 21·24	CMED

Note: [1]Price considering 500 mg tablets (30 tablets package); [2]Price considering 500 mg/mL (20 mL vial); [3]Values considering a mean body weight of 25 Kg; [4] Values assessed on February, 2023. CMED: Drug market regulation chamber council (PMC 18%: Maximum Consumer Price, 18% taxes).

Table S 5. Productivity loss costs (BRL) due to outpatient period among patients with dengue, categorized by age range and gender in Brazilian cities.

Source: Based on Information for the Public Employment and Income System (ISPER)

Age range (Years)	Niterói		São Paulo		Belo Horizonte		Goiânia		Campo Grande		Fortaleza		Manaus	
	**Male**	**Female**	**Male**	**Female**	**Male**	**Female**	**Male**	**Female**	**Male**	**Female**	**Male**	**Female**	**Male**	**Female**
0 to 17	847·79	1169·27	1169·27	1100·52	1100·52	1023·29	1023·29	980·09	980·09	744·78	744·78	837·45	837·45	847·79
18 to 24	398·81	559·76	514·1	484·79	431·37	420·73	430·13	446·99	419·44	370·25	352·58	438·02	412·34	398·81
25 to 29	559·97	933·68	864·7	804·64	708·62	630·29	649·88	679·42	643·47	503·26	495·27	621·32	591·38	559·97
30 to 39	796·76	1390·33	1235·4	1232·65	1070·92	937·29	989·33	1047·68	943·43	717·38	702·32	922·88	839·33	796·76
40 to 49	955·49	1637·34	1407·65	1517·18	1312·65	1273·14	1303·53	1361·57	1186·98	875·4	873·41	1133·69	950·24	955·49
50 to 64	1173·76	1659·03	1413·94	1678·68	1414·41	1413·93	1459·82	1610·12	1361·52	1113·4	1119·87	1353·61	1118·18	1173·76
65 to 66	847·79	1954·3	1169·27	2014·05	1100·52	1770·68	1023·29	1860·14	980·09	1922·24	744·78	1638·04	837·45	847·79
66 or more	847·79	1169·27	1169·27	1100·52	1100·52	1023·29	1023·29	980·09	980·09	744·78	744·78	837·45	837·45	847·79
**Overall**	852·24	1360·83	1171·6	1286·2	1085·77	1064·54	1065·66	1171·81	1001·16	822·98	779·68	1007·11	849·25	852·24

Table S 6. Productivity loss costs (BRL) due to hospitalization period among patients with dengue, categorized by age range and gender in Brazilian cities.

Age range (years)	Niterói		São Paulo		Belo Horizonte		Goiânia		Campo Grande		Fortaleza		Manaus	
	**Male**	**Female**	**Male**	**Female**	**Male**	**Female**	**Male**	**Female**	**Male**	**Female**	**Male**	**Female**	**Male**	**Female**
0 to 17	1829·44	1829·44	2523·15	2523·15	2374·81	2374·81	2208·15	2208·15	2114·94	2114·94	1607·16	1607·16	1807·14	1807·14
18 to 24	997·71	860·59	1207·9	1109·37	1046·12	930·86	907·88	928·17	964·57	905·11	798·97	760·83	945·2	889·79
25 to 29	1638·62	1208·37	2014·79	1865·93	1736·32	1529·12	1360·1	1402·37	1466·12	1388·53	1085·99	1068·74	1340·74	1276·14
30 to 39	2344·19	1719·31	3000·18	2665·86	2659·92	2310·94	2022·57	2134·88	2260·79	2035·83	1548·03	1515·53	1991·47	1811·19
40 to 49	2562·9	2061·85	3533·21	3037·56	3273·91	2832·56	2747·3	2812·88	2938·12	2561·38	1889·03	1884·72	2446·38	2050·51
50 to 64	2404·89	2532·85	3580·01	3051·14	3622·41	3052·14	3051·12	3150·13	3474·47	2938·03	2402·61	2416·57	2920·94	2412·92
65 to 66	3090·36	1829·44	4217·18	2523·15	4346·1	2374·81	3820·94	2208·15	4013·99	2114·94	4147·99	1607·16	3534·72	1807·14
66 or more	997·71	1829·44	2523·15	2523·15	2374·81	2374·81	2208·15	2208·15	2114·94	2114·94	1607·16	1607·16	1807·14	1807·14
**Overall**	2124·78	1839·04	2936·54	2528·2	2775·49	2342·97	2297·17	2299·58	2528·65	2160·39	1775·89	1682·46	2173·23	1832·6

Source: Based on Information for the Public Employment and Income System (ISPER)

Table S 7. Productivity loss costs (BRL) due to premature mortality among patients with dengue, categorized by age range and gender in Brazilian cities.

Age at death (years)	Niterói		São Paulo		Belo Horizonte		Goiânia		Campo Grande		Fortaleza		Manaus	
	Male	Female	Male	Female	Male	Female	Male	Female	Male	Female	Male	Female	Male	Female
18	1141705·16	950111·32	1517176·87	1337605·37	1394092·39	1206235·91	1136426·68	1175979·81	1249624·9	1110246·64	874034·41	862697·36	1091981·22	961571·76
19	1171437·43	959269·86	1562187·5	1353072·73	1439137·54	1221935·8	1171908·04	1190765·55	1290525·9	1123480·24	898892·23	871531·54	1124244·74	970366·69
20	1195186·87	968725·58	1598955·71	1369041·99	1476549·86	1238145·14	1202017·53	1206031·07	1324929·09	1137143·24	920007·06	880652·36	1151147·51	979447·01
21	1227338·19	978488·12	1645658·92	1385529·42	1524126·68	1254880·44	1241024·51	1221791·92	1367652·21	1151249·59	954562·91	890069·14	1185851·23	988821·98
22	1242039·35	988567·44	1668640·94	1402551·84	1547239·2	1272158·77	1258431·75	1238064·19	1387740·93	1165813·67	965417·5	899791·48	1200528·4	998501·14
23	1257217·53	998973·82	1692368·69	1420126·61	1571101·68	1289997·76	1276403·83	1254864·47	1408481·49	1180850·33	976624·31	909829·3	1215681·82	1008494·38
24	1272888·22	1009717·87	1716866·37	1438271·65	1595738·46	1308415·59	1294959·07	1272209·89	1429895·05	1196374·91	988194·76	920192·83	1231326·95	1018811·88
25	1293207·63	1020810·55	1747941·16	1457005·47	1626594·28	1327431·05	1318003·72	1290118·14	1456666·53	1212403·23	1002851·86	930892·63	1251298·21	1029464·17
26	1289632·4	1023505·54	1747953·33	1457295·75	1630055·7	1331998·22	1322507·65	1296666·27	1462199·26	1216778·15	1005246·49	934186·02	1254196·47	1030733·25
27	1290215·73	1026287·97	1753935·73	1457595·44	1639224·9	1336713·59	1331171·18	1303426·87	1472725·91	1221295·02	1010518·01	937586·28	1261131·15	1032043·52
28	1·290·818,00	1029160·69	1760112·24	1457904·86	1648691·64	1341581·97	1340115·82	1310406·85	1483594·13	1225958·47	1015960·58	941096·87	1268290·85	1033396·3
29	1295316·05	1032126·63	1775935·91	1458224·31	1669347·17	1346608·31	1362194·87	1317613·31	1506819·14	1230773·23	1027623·15	944721·37	1283745·06	1034792·97
30	1292081·79	1035188·8	1773073·02	1458554·14	1668556·61	1351797·75	1358885·25	1325053·61	1506399·99	1235744·23	1027381·26	948463·49	1283314·75	1036234·97
31	1274977·19	1025483·82	1755056·72	1438750·97	1655717·37	1337468·12	1352047·12	1314289·66	1498349·68	1224576·62	1021736·12	941075·93	1274807·66	1024250·37
32	1257317·58	1015463·92	1736455·81	1418305·23	1642461·52	1322673·5	1344987·1	1303176·43	1490038·15	1213046·65	1015907·81	933448·66	1266024·53	1011876·9
33	1239084·94	1005118·89	1717251·34	1397196·06	1628775·54	1307398·83	1·337·698,00	1291702·6	1481456·93	1201142·54	1009890·39	925573·89	1256956·41	999101·92
34	1220260·68	994438·18	1697423·71	1375401·92	1614645·47	1291628·52	1330172·38	1279856·46	1472597·26	1188852·16	1003677·7	917443·6	1247594·04	985912·42
35	1200825·6	983410·9	1676952·71	1352900·61	1600056·9	1275346·49	1322402·56	1267625·93	1463450·1	1176162·99	997263·43	909049·5	1237927·87	972294·94
36	1180759·89	972025·8	1655817·46	1329669·16	1584994·96	1258536·13	1314380·63	1254998·54	1454006·14	1163062·07	990641·02	900383·02	1227948·05	958235·6
37	1160043·08	960271·27	1633996·4	1305683·89	1569444·28	1241180·31	1306098·4	1241961·42	1444255·73	1149536·04	983803·73	891435·33	1217644·41	943720·05
38	1138654·04	948135·33	1611467·29	1280920·34	1553389·01	1223261·31	1297547·42	1228501·26	1434188·94	1135571·12	976744·57	882197·3	1207006·43	928733·5
39	1116570·96	935605·6	1588207·14	1255353·24	1536812·77	1204760·87	1288718·97	1214604·34	1423795·5	1121153·06	969456·36	872659·51	1196023·26	913260·66
40	1093771·32	922669·3	1564192·24	1228956·54	1519698·66	1185660·13	1279604·06	1200256·48	1413064·81	1106267·16	961931·66	862812·24	1184683·7	897285·75
41	1064724·26	900687·53	1525975·58	1192343·37	1486567·95	1152804·18	1251943·61	1168369·92	1384929·72	1077663·89	945575·96	843348·78	1161520·94	874765·97
42	1034734·67	877992·49	1486518·85	1154542·16	1452362·19	1118882·1	1223385·62	1135448·69	1355881·7	1048132·5	928689·55	823253·76	1137606·58	851515·46
43	1003771·97	854561·03	1445781·82	1115514·37	1417046·52	1083859·31	1193900·97	1101459·22	1325891·11	1017642·86	911255·19	802506·69	1112916·23	827510·52

44	971804·58	830369·26	1403722·94	1075220·18	1380584·91	1047700·09	1163459·6	1066366·85	1294927·38	986163·88	893255·13	781086·41	1087424·73	802726·65
45	938799·9	805392·51	1360299·31	1033618·51	1342940·19	1010367·56	1132030·45	1030135·78	1262958·92	953663·46	874670·99	758971·07	1061106·07	777138·59
46	904724·27	779605·3	1315466·66	990666·94	1304073·95	971823·65	1099581·47	992729·08	1229953·14	920108·46	855483·82	736138·13	1033933·41	750720·23
47	869542·94	752981·34	1269179·26	946321·66	1263946·56	932029·05	1066079·58	954108·59	1195876·38	885464·65	835674·06	712564·3	1005879·04	723444·64
48	833220·04	725493·47	1221389·91	900537·44	1222517·11	890943·18	1031490·61	914234·93	1160693·88	849696·7	815221·51	688225·54	976914·35	695·284,00
49	795718·51	697113·67	1172049·87	853267·6	1179743·34	848524·14	995779·28	873067·43	1124369·76	812768·14	794105·31	663097·02	947009·81	666209·6
50	757000·13	667812·99	1121108·83	804463·94	1135581·62	804728·68	958909·17	830564·11	1086866·99	774641·31	772303·91	637153·12	916134·91	636191·78
51	721004·42	625700·98	1067336·41	753734·78	1081210·96	753982·83	913191·93	778189·09	1034641·12	725792·76	736862·27	596974·52	872307·86	596073·8
52	683840·7	582222·52	1011819·16	701359·55	1025076·06	701590·36	865991·24	724114·59	980720·61	675359·15	700270·61	555492·18	827058·69	554654·05
53	645471·09	537333·24	954500·47	647284·83	967119·66	647497·84	817258·96	668285·46	925050·47	623289·06	662491·6	512663·81	780341·26	511890·3
54	605856·44	490987·38	895321·87	591455·47	907282·68	591650·11	766945·4	610644·76	867573·92	569529·37	623486·73	468445·73	732107·93	467738·94
55	564956·36	443137·68	834223·03	533814·54	845504·09	533990·21	714999·24	551133·72	808232·34	514025·27	583216·21	422792·85	682309·5	422154·94
56	522729·14	393735·33	771141·63	474303·25	781720·88	474459·34	661367·52	489691·64	746965·23	456720·15	541638·98	375658·6	630895·2	375091·81
57	479131·72	342729·95	706013·34	412860·92	715868·01	412996·79	605995·53	426255·86	683710·11	397555·58	498712·64	326994·92	577812·58	326501·55
58	434119·63	290069·53	638771·75	349424·89	647878·32	349539·88	548826·81	360761·69	618402·45	336471·21	454393·4	276752·19	523007·52	276334·63
59	387646·97	235700·37	569348·27	283930·46	577682·47	284023·89	489803·07	293142·35	550975·67	273404·75	408636·08	224879·16	466424·12	224539·86
60	339666·34	179567·01	497672·11	216310·84	505208·89	216382·02	428864·09	223328·86	481·361,00	208291·88	361394·01	171322·94	408004·69	171064·44
61	290128·83	121612·22	423670·18	146497·07	430383·65	146545·28	365947·75	151250·05	409487·44	141066·21	312619·01	116028·9	347689·64	115853·83
62	238983·9	61776·89	347267·01	74417·96	353130·46	74442·45	300989·87	76832·39	335281·71	71659·18	262261·34	58940·66	285417·46	58851·73
63	186179·4	0	268384·67	0	273370·52	0	233924·21	0	258668·12	0	210269·64	0	221124·65	0
64	131661·49	0	186942·74	0	191022·51	0	164682·38	0	179568·54	0	156590·9	0	154745·65	0
**65**	75374·55	0	102858·14	0	106002·43	0	93193·77	0	97902·31	0	101170·37	0	86212·75	0
**Overall**	908794·62	728752·91	1274883·15	980996·01	1227714·14	927595·36	1018255·18	925418·34	1131736·41	862637·33	788304·51	676272·52	961567·92	717991·86

Source: Based on Information for the Public Employment and Income System (ISPER)

Table S 8. Hospitalization rate of suspected dengue cases in Brazilian cities during 2008 to 2019.

Age range (years)	Niterói	São Paulo	Belo Horizonte	Goiânia	Campo Grande	Fortaleza	Manaus
<1	8·92%	8·35%	4·42%	7·32%	4·1%	6·16%	5·52%
01 to 04	4·98%	7·31%	3·72%	5·45%	2·77%	6·51%	3·96%
05 to 09	5·66%	6·18%	2·7%	4·96%	2·56%	6·16%	5·7%
10 to 19	3·41%	3·95%	1·1%	2·3%	1·84%	4·59%	4·84%
20 to 39	1·59%	3·65%	0·83%	1·94%	1·65%	1·45%	2·3%
40 to 59	1·85%	4·4%	1·16%	3·22%	2·28%	2·03%	2·79%
60 or more	2·86%	7·75%	2·61%	5·81%	5·06%	3·95%	3·29%
**Overall**	**4·18%**	**5·94%**	**2·36%**	**4·43%**	**2·89%**	**4·41%**	**4·06%**

Source: SINAN, Ministry of Health.

Table S 9. Severe rates of dengue hospitalizations in Brazilian cities during 2016 to 2019.

Age range (years)	Niterói	São Paulo	Belo Horizonte	Goiânia	Campo Grande	Fortaleza	Manaus
00 to 19	20·89%	6·76%	13·98%	17·77%	23·25%	12·52%	5·25%
20 to 39	9·39%	8·2%	14·6%	18·99%	26·75%	5·65%	31·75%
40 to 59	22·15%	3·91%	8·59%	13·55%	18·12%	10·28%	26·29%
60 to 79	9·62%	5·6%	9·61%	15·29%	22·07%	7·06%	21·74%
80 or more	25,00%	9·09%	5·99%	13·51%	25,00%	9·86%	15,00%
**Overall**	**17·41%**	**6·71%**	**10·55%**	**15·82%**	**23·04%**	**9·07%**	**20,00%**

Note: Nonclassic cases include hemorrhagic fever. Source: SINAN, Ministry Of Health.

Table S 10. Case fatality rate of dengue hospitalizations in Brazilian cities during 2016 to 2019.

Age range (years)	Niterói	São Paulo	Belo Horizonte	Goiânia	Campo Grande	Fortaleza	Manaus
00 to 19	1·27%	1·08%	0·82%	1·03%	0·54%	0·78%	0·13%
20 to 39	0·16%	0·3%	0·12%	0·62%	0·53%	0·12%	0·2%
40 to 59	4·03%	1·71%	0·86%	0·48%	0·65%	2·34%	0·23%
60 to 79	3·85%	2·28%	0·7%	2·13%	1·19%	4·28%	0·87%
80 or more	2·33%[1]	3·64%	1·76%	3·93%	4·55%	7·75%	0·36%[1]
**Overall**	2·33%	1·8%	0·85%	1·64%	1·49%	3·05%	0·36%

Note: [1] Imputation by mean. Source: SINAN, Ministry Of Health.

Table S 11. Direct and indirect costs according to the seven cities during the 20 years horizon

City	**Direct Costs** (per 1,000 people)			**Indirect Costs** (per 1,000 people)			**Total Cost difference**
	Without Wolbachia	With Wolbachia	Cost difference	Without Wolbachia	With Wolbachia	Cost difference	
Manaus	R$ 464,839·66	R$ 465,329·17	R$ 489·51	R$ 35,292,831·42	R$ 34,794,050·38	-R$ 498,781·04	-R$ 498,291·54
São Paulo	R$ 112,971·02	R$ 119,236·42	R$ 6,265·40	R$ 42,606,805·59	R$ 41,994,511·20	-R$ 612,294·39	-R$ 606,028·99
Fortaleza	R$ 515,983·24	R$ 460,447·58	-R$ 55,535·66	R$ 31,266,706·41	R$ 29,901,092·88	-R$ 1,365,613·53	-R$ 1,421,149·19
Niterói	R$ 629,599·80	R$ 531,100·38	-R$ 98,499·41	R$ 40,307,600·26	R$ 38,759,841·05	-R$ 1,547,759·21	-R$ 1,646,258·62
Belo Horizonte	R$ 697,927·88	R$ 505,042·90	-R$ 192,884·98	R$ 45,537,207·19	R$ 41,080,911·58	-R$ 4,456,295·61	-R$ 4,649,180·59
Campo Grande	R$ 761,810·73	R$ 562,756·25	-R$ 199,054·48	R$ 43,708,352·63	R$ 39,745,439·71	-R$ 3,962,912·92	-R$ 4,161,967·40
Goiânia	R$ 896,968·33	R$ 597,670·63	-R$ 299,297·70	R$ 46,031,322·21	R$ 40,475,317·43	-R$ 5,556,004·79	-R$ 5,855,302·49
**Overall (mean)**	**R$ 582,871·52**	**R$ 463,083·33**	**-R$ 119,788·19**	**R$ 40,678,689·39**	**R$ 38,107,309·18**	**-R$ 2,571,380·21**	**-R$ 2,691,168·40**

Table S 12. Scenario analysis considering the Health sector and Public Health system perspective and the implementation strategy of Wolbachia method compared to the common dengue control program strategies in Brazilian cities during 2023 to 2042.

**City**	**Cost difference** (per 1·000 people, BRL)	**DALY averted** (per 1·000 people)	**ICER** (BRL/DALY)	**NMB** (BRL)
Health sector				
Manaus	489·51	0·4	1,359·72	42·71
São Paulo	6,265·40	5·3	1,177·78	632·09
Niterói	- 98,499·41	14·6	Absolute dominance	1,852·42
Fortaleza	- 55,535·66	6·2	Absolute dominance	800·94
Belo Horizonte	- 192,884·98	3·3	Absolute dominance	594·23
Campo Grande	- 199,054·48	8·6	Absolute dominance	1,233·72
Goiânia	- 299,297·70	9·5	Absolute dominance	1,439·95
Public Health system				
Manaus	1,826·03	0·4	5,072·21	41·37
São Paulo	9,294·06	5·3	1,747·11	629·07
Niterói	- 87,363·40	14·6	Absolute dominance	1,841·28
Fortaleza	- 51,632·78	6·2	Absolute dominance	797·04
Belo Horizonte	- 182,194·97	3·3	Absolute dominance	583·54
Campo Grande	- 187,213·32	8·6	Absolute dominance	1,221·88
Goiânia	- 276,804·08	9·5	Absolute dominance	1,417·46
Implementation strategy (WMP team)				
Manaus	-R$ 479,497·82	0·4	Absolute dominance	R$ 522·70
São Paulo	-R$ 604,566·38	5·3	Absolute dominance	R$ 1,242·93
Fortaleza	-R$ 1,329,902·84	6·2	Absolute dominance	R$ 5,089·89
Niterói	-R$ 2,288,496·62	13·6	Absolute dominance	R$ 3,916·77
Belo Horizonte	-R$ 4,626,000·59	3·3	Absolute dominance	R$ 5,027·35
Campo Grande	-R$ 4,138,787·40	8·6	Absolute dominance	R$ 5,173·45
Goiânia	-R$ 5,832,122·49	9·5	Absolute dominance	R$ 6,972·77

Note: DALY: Disability-adjusted life years; ICER: Incremental cost-effectiveness ratio: NMB: Net Monetary Benefits (incremental)
