# Supplementary material for: Simulation-based economic evaluation of the Wolbachia method in Brazil: a cost-effective strategy for dengue control
Source: Lancet Reg Health Am. 2024 Jun 3;35:100783. doi: 10.1016/j.lana.2024.100783 (PMC11190723; doi:10.1016/j.lana.2024.100783)
Supplement: Translated_Summary_Portugues [file mmc2.docx]

***Editorial Disclaimer:*** *This translation in Portuguese was submitted by the authors and we reproduce it as supplied. It has not been peer-reviewed. Our editorial processes have only been applied to the original abstract in English, which should serve as a reference for this manuscript.*

**Resumo**

**Contexto**: O vírus da dengue (DENV) é um arbovírus transmitido pelo mosquito *Aedes aegypti*, podendo causar condições graves como febre hemorrágica e síndrome de choque da dengue. Essas condições estão associadas a consequências sociais, clínicas e econômicas adversas no Brasil. Neste contexto, o método de substituição do mosquito por species infectadas com a bactéria Wolbachia surge como uma estratégia promissora de controle da dengue.

**Métodos**: Estimamos o impacto econômico da implementação do método Wolbachia em sete diferentes cidades brasileiras. Um modelo matemático de microssimulação acompanhou cerca de 23 milhões de habitantes ao longo de um período de 20 anos, considerando as transições entre cinco diferentes estados de saúde (suscetível, infecção inaparente, quadro ambulatorial, hospitalização e morte). Os custos diretos incluíram os recursos envolvidos nos programas locais de controle da dengue, implementação de Wolbachia e cuidados com o tratamento da dengue. Custos indiretos relacionados à morte premature e perda de produtividade, bem como os anos de vida ajustados por incapacidade (DALY) evitados também foram considerados.

**Resultados**: Sem Wolbachia, o modelo projetou 1.762.688 casos de dengue notificados ao longo de 20 anos. A implementação do método Wolbachia poderia evitar pelo menos 1.295.566 casos de dengue, resultando em custos menores e maior eficácia em todas as cidades simuladas. Em média, para cada 1.000 habitantes acompanhados por 20 anos, o método Wolbachia gerou uma diferença de custo de USD 538.233,68 (BRL 2.691.168,40) e evitou 5,56 DALYs. Os benefícios monetários líquidos (NMB) foram positivos em todas as sete cidades, variando de USD 110,72 (BRL 553,59) a USD 1.399,19 (BRL 6.995,95) por habitante. A análise de sensibilidade também mostrou um retorno favorável sobre o investimento com uma relação benefício-custo (BCR) positiva.

**Interpretação**: Wolbachia é provavelmente uma estratégia custo-efetiva no contexto brasileiro, consistente com estudos internacionais. Análises de sensibilidade e cenários alternativos confirmaram a robustez dos resultados.

**Financiamento**: Este estudo recebeu uma bolsa do Programa Mundial de Mosquitos (WMP).
